# The Expanding Clinical Spectrum of Myelin Oligodendrocyte Glycoprotein (MOG) Antibody Associated Disease in Children and Adults

**DOI:** 10.3389/fneur.2020.00960

**Published:** 2020-09-09

**Authors:** Erica Parrotta, Ilya Kister

**Affiliations:** ^1^Saint Peter's Health Partners, Saint Peter's MS & Headache Center, Albany, NY, United States; ^2^New York University Langone Medical Center, Multiple Sclerosis Comprehensive Care Center, New York, NY, United States

**Keywords:** MOG (myelin oligodendrocyte glyco protein), MOG antibody disease, ADEM, myelitis, optic neuritis, CRION, brainstem encephalitis

## Introduction

The ability of MOG antibody (MOG-Ab) to induce autoimmune disease in animals has been known for decades (1), but it is only recently since the cell-based assay for MOG-Ab IgG_1_ has been developed and commercialized, that it became possible to characterize clinical syndromes associated with MOG-Ab in humans. Early reports of MOG Associated Disease (MOGAD) emphasized its similarity to Neuromyeliits Optica Spectrum Disorder (NMOSD) (2–4). Indeed, a minority of patients with Aquaporin-4 antibody (AQ4-ab)-seronegative NMOSD−42% in one series–test positive for MOG-Ab (5). However, because the spectrum of MOGAD encompasses many NMOSD-atypical presentations, and because of differences in pathophysiology–AQ4-ab-positive NMOSD being an astrocytopathy and MOGAD being an oligodendrocytopathy—there is an increasing tendency to recognize AQ4-Ab-positive NMOSD and MOGAD as distinct entities (6–10).

In this review, we organize the clinical presentations of MOGAD by neuroanatomic compartments, while emphasizing the wide range of reported presentations. While this organization is useful for didactic purposes, it should be borne in mind that MOGAD may involve multiple regions of the CNS simultaneously– much more often than other CNS inflammatory diseases, and that half of MOGAD patients have active lesions in more than one location at the time of initial presentation (11–14).

While no phenotype is restricted to any specific age group, some generalizations about clinical presentations of MOGAD in children and adults are possible. In children under the age of 11, ADEM-like phenotypes (encephalopathy, multifocal neurologic deficits and “fluffy” supratentorial cerebral lesions in a bilateral distribution) predominate, while in adolescents and adults, focal syndromes of optic neuritis or longitudinally extensive myelitis are more common (11, 15, 16). Unlike Multiple Sclerosis (MS), where relapse rates are higher in children and decline with older age, in MOGAD the majority of children are not prone to frequent relapses, with 80% of having a monophasic course (17). However, the high rate of monophasic disease may be an overestimate due short follow up (right censoring) as recent case reports documented disease reemergence years and even decades after the initial episode in childhood (18, 19). Given the important differences in pediatric and adult MOGAD, we will qualify discussion of specific syndromes with reference to the respective age group (with the caveat that the clinical distinctions across age groups are only generalizations).

## Optic Neuritis and Other Visual Pathway Presentations

Optic neuritis (ON) is the most common initial presentation of MOGAD in adolescence and adulthood, and a frequent presentation in pediatric patients (11, 16, 20). It is associated with a higher risk of subsequent relapse compared to other clinical presentations (11–13, 18). At the onset, vision loss is often severe and up to 80% of patients have bilateral optic nerve involvement, which is highly unusual in MS (12, 14, 21–24). Despite the severity of vision loss in the acute phase, recovery is usually good, especially in children: 89–98% of children had visual acuity to 20/25 or better at 6 months (14, 25). In adults, 6–14% of patients had permanent loss of vision (≤ 20/200) in the affected eye (11, 13, 24).

Optic disc edema is rare in MS or NMOSD but is present in up to 86% of patients with MOGAD-ON (13, 21, 22, 24, 26, 27). Rarely, bilateral ON with disc edema can be mistaken for idiopathic intracranial hypertension especially if the patient also complains of headache and has elevated opening pressure on lumbar puncture; however lymphocytic pleocytosis in CSF and enhancement of optic nerve on orbital MRI point toward an inflammatory etiology and should prompt testing for MOG-Ab (28). Fulminant disc edema with peripapillary hemorrhages and “macular star” have been described in MOGAD-ON (29–31). Both of these findings are considered highly atypical for other inflammatory-demyelinating diseases and are more often associated with infectious and ischemic etiologies (29, 30).

Up to 50% of adults with MOG-ON have a recurrence of optic neuritis (11–13, 18), which may be the only manifestations of MOGAD. Two rare previously described phenotypes, chronic relapsing inflammatory optic neuropathy (CRION)– a rare condition characterized by relapsing, steroid-dependent optic neuritis (32), and relapsing isolated optic neuritis (RION), have been associated with MOG-Ab in some cases (33, 34).

**Figure 1:**
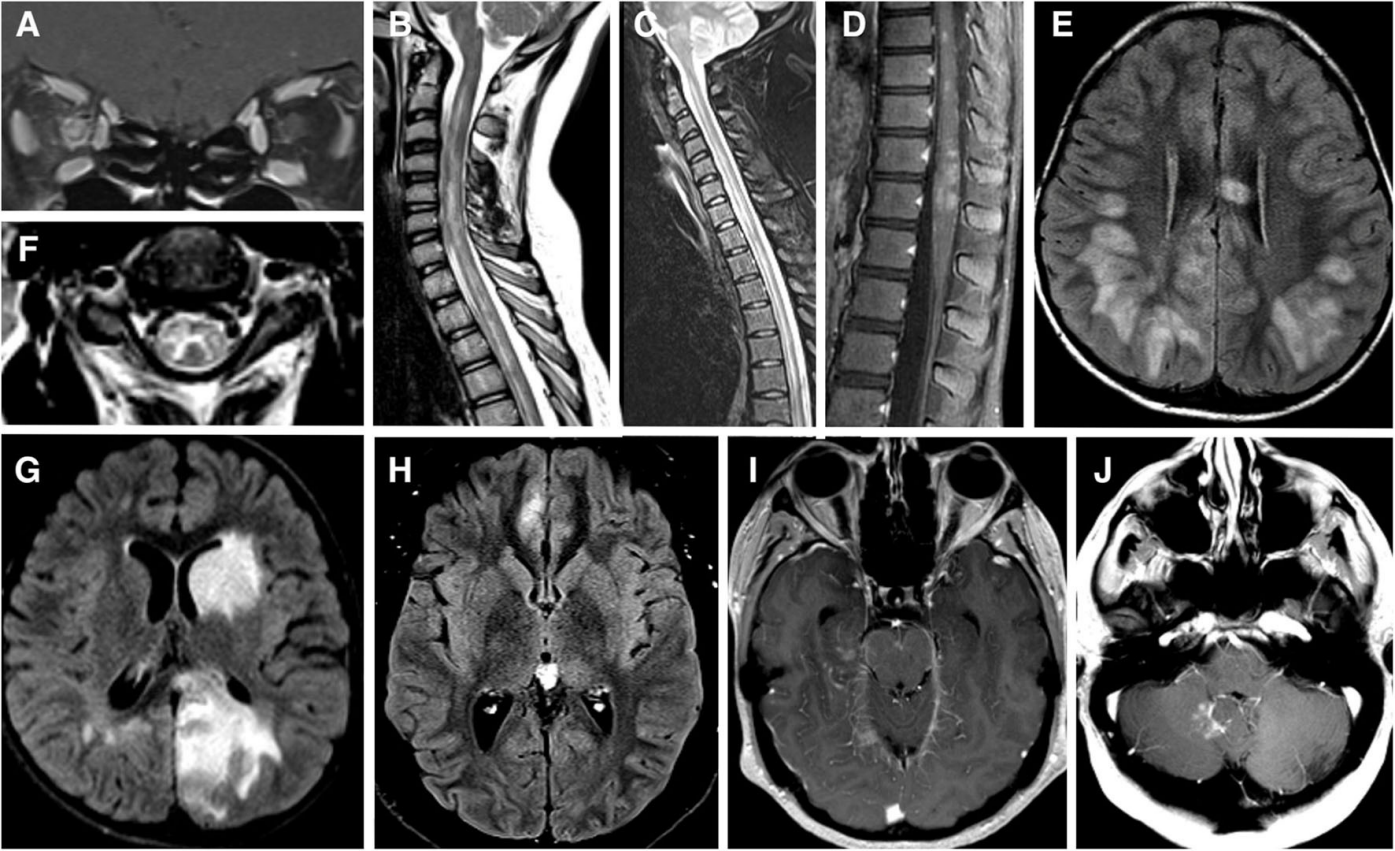
**(A)** MRI brain T1 coronal post gadolinium contrast showing contrast enhancement of bilateral optic nerves and right optic nerve sheath consistent with perioptic neuritis. **(B)** MRI spine sagittal STIR showing longitudinal extensive patchy lesion spaning from cervical to thoracic cord. **(C)** MRI spine sagittal T2 showing hyperintense longitudinally extensive “pseudo-dilation” of central canal. **(D)** MRI spine sagittal T1 post gadolinium contrast showing patchy enhancement of the conus medullaris. **(E)** MRI brain axial FLAIR showing large subcortical and septal white matter lesions in a pediatric patient presenting with ADEM. **(F)** MRI brain axial T2 with hyperintense “H” sign outlining the central gray matter of the upper cervical cord in a teenager with myelitis. **(G)** MRI brain axial T2 with “fluffy” hyperintense lesion of gray and white matter of the left caudate and left occipital parietal regions in a pediatric patient who presenting with ADEM. **(H)** MRI brain axial T2 showing unilateral FLAIR hyperintensity and edema of right mesial frontal cortex in a patient with FLAMES syndrome. **(I)** MRI brain axial T1 post gadolinium contrast showing leptomeningeal enhancement of the midrain and right mesial temporal lobe. **(J)** MRI brain axial T1 post gadolinium contrast showing a lesion adjacent to the cerebellar vermis and dorsal medulla in a patient with brainstem syndrome and no other lesions.

MRI of the orbits during acute MOG-ON typically shows longitudinally extensive optic nerve enhancement with a predilection for the anterior portion of optic nerves; the chiasm and optic tracts are less frequently affected (21, 31). “Optic perineuritis,” characterized by inflammation of the optic nerve sheath and surrounding structures on MRI (35), is seen in up to 50% of cases of MOGAD-ON (Figure 1A) (13, 21, 25, 36, 37). Perineural enhancement is a feature that can help differentiate MOGAD from NMOSD or MS (13, 21, 25, 36, 37). Isolated cases of MOGAD perineuritis, involving the nerve sheath and surrounding structures but not the optic nerve, have also been reported (38, 39). Rarely, uveitis and keratitis can occur simultaneously or subsequently to MOG-ON (38).

## Transverse Myelitis

MOG-Ab associated acute transverse myelitis is a relatively common presentation of MOGAD in adults, and can be seen in children as well (11). In some cases of MOG-TM, there is an antecedent history of infection or vaccination, but in most patients, no such history can be elicited (11, 18, 40). While MOG-TM is typically steroid-responsive with favorable long-term recovery, around 9% of patients have poor recovery (11). Recurrent myelitis, without any other syndromes of MOGAD, is reported in up to 5% of patients (41).

MOG-TM can affect any segments of the spinal cord but has a greater predilection for conus medullaris–reported in 11–41% patients–than other CNS inflammatory-demyelinating diseases (11, 18, 40, 42). The involvement of the conus (Figure 1D) may explain the high incidence of neurogenic bowel and bladder symptoms (83%), and erectile dysfunction (54%) during acute phase (40), as well as in the long-term (11). There are also reports of a steroid-dependent myeloradiculitis in MOGAD with a longitudinally extensive transverse lesion from T12 to the conus with sacral nerve root enhancement (43).

Radiographically, MOG-TM is usually associated with a longitudinally extensive lesion spanning 3-4 vertebral segments (Figure 1B) (2, 18, 40, 44). In this respect, MOG-TM is similar to NMO-TM, but there are several radiographic differences between the two diseases. First, cord lesion of MOG-TM during the acute phase are much less likely to demonstrate gadolinium enhancement than in NMOSD: only 26% of MOG patients show enhancement vs. 78% of AQ4-ab-seropositive NMOSD (40). Secondly, spinal cord lesions in MOGAD can be multifocal: 62% of patients had ≥2 non-contiguous spinal cord lesions (40). The radiographic multifocality is in line with the notion that MOGAD has a tendency to affect multiple areas of CNS simultaneously.

MOG-TM affects both gray and white matter of the cord. The involvement of gray matter can manifest as linear hyperintensity of the central spinal canal (“pseudo-dilation,” Figure 1C) (44), or as H-shaped T2-hyperintensity that outlines the anterior and posterior horns (“H-sign,” Figure 1F) (2, 18, 40). The “H-sign” is suggestive, but not specific for MOGAD, reported in 29% of patients with MOG-TM and 8% of patients with NMO-TM (40). The predilection for the gray matter may explain why MOG-TM sometimes presents as acute flaccid paralysis (AFM) (45): in one series 10 out of 47 MOGAD patients (21%) met clinical criteria for AFM (40).

## Acute Disseminated Encephalomyelitis (ADEM) and Other Cerebral Presentations

In young children, MOGAD frequently presents as ADEM or an ADEM-like syndrome (ADEM with optic neuritis, multiphasic disseminated encephalomyelitis) (16, 46–49). MRI of the brain typically shows large, ill-defined bilateral lesions frequently involving cortical and deep gray matter structures (Figure 1G) (50). Lesions may also involve subcortical white matter and corpus callosum as seen in Figure 1E. Optic nerves and spinal cord may be involved concurrently with brain (51). Recurrent ADEM or ADEM associated with recurrent optic neuritis (52, 53) are especially suggestive of MOGAD. Importantly, in children with clinical syndrome of encephalitis, MOGAD diagnosis is possible even when MRI findings are not compatible with ADEM—for example, exclusive cortical or symmetric thalamic/basal ganglia involvement, or even normal MRI (54).

Cerebral involvement in adults is both less common and more restricted than in children, though there are exceptions (55). Syndrome of encephalitis with steroid-responsive seizures, also termed FLAMES (FLAIR-hyperintense Lesions and Anti-MOG-associated Encephalitis with Seizures), appears to be specific to MOGAD (20, 56–58). FLAMES patients present with focal-onset, tonic-clonic seizures, and have unilateral FLAIR hyperintensities with edema on MRI (Figure 1H). A review by Budhram et al. found 20 cases of FLAMES in the literature. The most common symptoms were seizures (85%), headache (70%), and fever (55%). CSF pleocytosis and cortical leptomeningeal enhancement (Figure 1I) were present in a minority of patients (57). All patients with FLAMES responded to high dose steroids with resolution of FLAIR changes. Of note, a number of patients developed ON either before or after seizures (56, 58, 59). Thus, the emergence of seizures in the context of ON or focal brain inflammatory lesions should prompt testing for MOG-Ab (52).

Isolated seizures may rarely be an index event in MOGAD. In one case, an adult patient presented with aphasic status epilepticus with initial MRI showing no abnormalities. Six months later the patient developed a tumefactive demyelinating lesion, with MOG-Ab testing positive several months later (60). A similar presentation has been described in four pediatric patients who presented with isolated seizures and normal brain MRI and developed MRI brain lesions months, and in one case years, later (61).

Several studies document an association between MOGAD and autoimmune encephalitis with NMDA-antibody (62–64). In a retrospective case review by Titulaer et al., 12 of 691 with NMDAR encephalitis patients (1.6%) tested positive for MOG-Ab. Some patients presented with MOGAD syndrome followed by encephalitis, others with encephalitis followed by MOGAD, and in some NMDA encephalitis and MOGAD were diagnosed concurrently. Three patients with NMDAR encephalitis and no clinical or MRI features to suggest MOGAD also tested positive for MOG-Ab (62).

Finally, mention should be made of rare cases when MOG-Ab was found in patients with pathologically-proven CNS vasculitis (65, 66). Two patients presented with fever, headache, confusion, and focal neurologic deficits (66), and the third had 9 months of progressive cognitive and behavioral decline (65). MRI showed multifocal lesions in both the gray and white matter in two cases, one of whom also had open-ring contrast-enhancing lesions. The third case had findings of focal cortical encephalitis with gyriform FLAIR hyperintensities with edema, similar to findings seen in FLAMES. All three cases underwent brain biopsy, which showed small vessel perivascular inflammation, consistent with CNS vasculitis. However, fibrinoid necrosis, a pathologic requirement for small vessel CNS vasculitis, was absent in two of the cases (66, 67). Whether vasculitis should be regarded as a primary or secondary manifestation of MOGAD, or MOG-Ab is unrelated to vasculitis diagnosis, is difficult to determine given rarity of the association.

## Brainstem and Cerebellar Presentations

Brainstem involvement is seen in 30% of MOGAD patients, and is a risk factor for a higher disability at long-term follow-up and more active disease (68). In one large series brainstem inflammation occurred concomitantly with inflammation in optic nerves in 40% of cases, spinal cord in 89% cases and cerebrum in 66% of cases (68). However, there are reports of isolated brainstem inflammation as well (Figure 1J) (68). Any part of the brainstem can be affected, medulla being the most common (11, 68). Brainstem lesions are usually associated with disabling symptoms—weakness, cranial nerve deficits, ataxia, hypoventilation syndrome, impaired consciousness and, and, exceptionally, a fatal outcome (68). Area postrema syndrome (APS), one of the core syndromes of NMOSD, has also been described in MOGAD (11, 68–70).

MOGAD can mimic infective rhomboencephalitis when a patient presents with fever, CSF leukocytosis, brainstem enhancing lesions and leptomeningeal enhancement (44, 68), or Chronic Lymphocytic Inflammation with Pontine Perivascular Enhancement Responsive to Steroids (CLIPPERS), when MRI shows punctate, curvilinear enhancement in the pons (71–73). Whether CLIPPERS is a form of MOGAD or elicits an immune response to MOG-Ab is uncertain (73).

## Conclusion

Since the first reports of MOG-Ab associated neurologic diseases appeared just a few years ago (4), the floodgates of case reporting have been opened and our understanding of MOGAD has grown exponentially. We now recognize certain clinical and radiologic features that help to differentiate MOG-ON and MOG-TM from NMOSD syndromes; that pediatric ADEM is frequently associated with MOG-Ab, especially if followed by episodes of ADEM or ON; that in adults, MOG can be associated with seizures and focal cerebral edema (“FLAMES syndrome,” which appears to be unique to MOGAD); that brainstem inflammation is seen in a significant minority of MOGAD patients and may be an isolated finding; that MOG Ab is a common mimicker of infectious encephalitis (54) that MOG antibody is exceptionally rare in MS or AQ4 Ab positive NMOSD, but may co-exist with NMDA and other autoimmune encephalidites (64, 74). But many important questions remain. We need to determine sensitivity, specificity, positive and negative predictive value of MOG-Ab in the various neurologic syndromes; whether MOG-Ab shoud be tested in CSF, if it is negative in serum (75); whether various ultrarare presentions, such as isolated seizures without brain lesions, CLIPPERS, and a MOG-Ab-associated CNS vasculitis-type syndrome should be subsumed under MOGAD rubric. Most importantly, we need to better stratify risk of disease recurrence after the first or second episode and determine best treatments to prevent recurrence. With the rapid pace of progress, we can expect to answer these and other questions, and, no doubt, find new surprises along the way.

## Author Contributions

EP and IK wrote sections of the manuscript. All authors contributed to manuscript revision, read and approved the submitted version.

## Conflict of Interest

IK served on advisory boards for Biogen and Genentech and received consulting fees from Roche and research support for investigator-initiated grants from Sanofi Genzyme, Biogen, EMD Serono, National MS Society, and Guthy Jackson Charitable Foundation. The remaining author declares that the research was conducted in the absence of any commercial or financial relationships that could be construed as a potential conflict of interest.
